# Development and construct validation of a parent-proxy quality of life instrument in children with bronchopulmonary dysplasia aged 4–8 years old

**DOI:** 10.1007/s11136-018-2029-7

**Published:** 2018-10-22

**Authors:** Lysbert Meijer-Schaap, Anthony E. J. Dubois, Boudewijn J. Kollen, Jet Tijmens-van der Hulst, Bertine M. J. Flokstra-de Blok, Elianne J. L. E. Vrijlandt

**Affiliations:** 1 Department of Pulmonary Diseases and Tuberculosis, University Medical Center Groningen, University of Groningen, Hanzeplein 1, Postbus 30.001, 9700 RB Groningen, The Netherlands; 2 Department of Pediatrics, Division of Pediatric Pulmonology and Allergy, University Medical Center Groningen, University of Groningen, Groningen, The Netherlands; 3 GRIAC Research Institute, University Medical Center Groningen, University of Groningen, Groningen, The Netherlands; 4 Department of General Practice, University Medical Center Groningen, University of Groningen, Groningen, The Netherlands

**Keywords:** Bronchopulmonary dysplasia, Health-related quality of life, Instrument development, Children

## Abstract

**Purpose:**

Children with bronchopulmonary dysplasia often develop complications that affect them well into adult life. Very little is known about how this affects their quality of life, since no sensitive instrument is available to measure health-related quality of life in this population. In this study, a Dutch parent-proxy instrument was developed for this purpose.

**Methods:**

A list of items was generated after literature search and interviews with both parents of patients and clinical experts. Clinically relevant items were selected with the clinical impact method and item analysis. Results of clinical tests to measure complications in children with bronchopulmonary dysplasia were correlated with these items to select the items that show construct validity. Cronbach’s alpha was calculated to estimate internal consistency of the items in the final questionnaire.

**Results:**

In total, 92 children and their parents and 7 clinicians participated. Of 130 identified items, 47 showed clinical relevance. Spirometry, the Child Behavior Checklist, mean arterial pressure, and body mass index were used to determine construct validity of 33 items. These items were structured within five domains: pulmonary complaints, school functioning, growth and nutrition, exercise and locomotion, emotional functioning and health care concerns. The questionnaire showed excellent internal consistency with Cronbach’s alpha of 0.919.

**Conclusion:**

This study developed a disease-specific parent-proxy instrument to measure health-related quality of life in children with bronchopulmonary dysplasia aged 4–8 years old, the BPD-QoL. All included items show construct validity and internal consistency reliability. Future research should focus on further validation and analysis of responsiveness and reliability.

**Electronic supplementary material:**

The online version of this article (10.1007/s11136-018-2029-7) contains supplementary material, which is available to authorized users.

## Introduction

Bronchopulmonary dysplasia (BPD) is a chronic lung disease that affects the mortality and morbidity of children born preterm [1, 2]. The pathology and definition of BPD is closely associated with low gestational age and low birth weight [3–5]. According to the National Institutes of Health Consensus definition, children are diagnosed with BPD when they are treated with more than 21% of oxygen at 28 days after birth. The severity of BPD is simultaneously graded as mild, moderate, or severe based on the respiratory support needed at discharge or at 36 weeks post menstrual age for children born < 32 weeks or 56 days postnatal age for children born ≥ 32 weeks gestational age (whichever comes first) [5]. The incidence of BPD found in large studies ranges from 25 to 68% in preterm children with a birth weight of less than 1500 g [6, 7]. Because of changing definitions and the developments in care for premature infants, it is difficult to determine the exact prevalence of BPD. It is estimated that about 10,000 children in the United States and about 500–2000 in The Netherlands fulfill the criteria for BPD [8, 9]. Several studies have shown that children who develop BPD experience more problems during childhood, adolescence, and adult age in comparison to children born preterm without BPD, as well as healthy controls [2, 10–12]. These problems emerge in different areas of functioning. Chest symptoms and pulmonary function abnormalities consist of airway obstruction, airway hyper reactivity, and hyperinflation as well as exercise restriction [1, 2, 11]. When compared to age-matched controls born at term, respiratory symptoms are more common and twice as many children with BPD have a diagnosis of asthma during childhood [8]. Because of increased vascular resistance in the lungs, children with BPD may develop pulmonary hypertension and right ventricular hypertrophy. Left ventricular hypertrophy is also seen, possibly associated with systemic hypertension which is more commonly found in children with BPD [13–15]. It has not been conclusively demonstrated that BPD is independently associated with neurodevelopmental problems, but it has been shown to be a significant risk factor. Children with BPD have a lower intelligence quotient (IQ) with 20% achieving full-scale IQ rates < 70. Of children 8 years old with BPD, 54% required special education [16]. Underdeveloped motor skills, both gross and fine, occur more often in patients with BPD compared to children with low birth weight without BPD and children born at term. More attention deficit problems are seen: at age 8 years old 15% of children with BPD was diagnosed with attention deficit hyperactivity disorder (ADHD), which was significantly more than children born at low birth weight (7%) and children born at term (4%) [16]. The degree of neurodevelopmental problems seems to be associated with the severity of BPD [5, 17]. Problems with growth and nutrition are common directly after birth, but are also seen later in childhood. The results of studies examining delay in growth at older age, however, are still inconclusive [18, 19].

Little is known about how these problems affect quality of life in children with BPD. Studies in school age children and young adults on the follow-up of BPD patients evaluating quality of life compared to either children born preterm without BPD or healthy controls show inconsistent results. Some studies report no significant difference [20–22], while other studies found evidence of lower HRQL [23, 24]. However, quality of life in all these studies was measured with a generic questionnaire. For health care purposes, disease-specific instruments to measure quality of life are known to be both more discriminative and responsive when compared to generic instruments and therefore are of greater clinical significance [25, 26]. For other respiratory conditions, such as asthma, cystic fibrosis, vocal cord dysfunction and sleep apnea, these instruments have been developed and found to be useful both in clinical practice and research [27]. There are specific challenges in measuring HRQL in children. When children are not able to complete self-report questionnaires or to understand the concept of quality of life, proxy-reports need to be used. There is no set age at which children are supposed to be able to self-report on quality of life. Of the self-report questionnaires on HRQL developed for children below 8 years of age only half of the instruments meet minimum standards for internal consistency [28–30]. Another important subject is that the perception of quality of life is related to age and development. This implies that an instrument must be developed and validated for a specified population. Currently, no disease-specific instrument to measure HRQL in children with BPD is available. Such an instrument would be very useful in long-term follow-up of these children since it would better address clinically important problems in this age group and consequently help to determine therapeutic goals. The aim of this study was to develop and validate a disease-specific parent-proxy questionnaire to measure health-related quality of life in Dutch children age 4–8 years old with BPD for future use in both research and clinical practice.

## Method

### Participant selection

The population consisted of 179 children aged 4–8 years old, who had been treated in the neonatal intensive care unit of the University Medical Center Groningen (UMCG) and met the criteria of BPD. This age group was selected because HRQL problems associated with BPD are expected to be experienced from school age. A proxy report instrument was chosen because the target population was 8 years or younger and also at increased risk of developmental delay. Children with relevant comorbidity that is not described as being related to BPD in literature were excluded [31]. Participants could only be included in the study if one of the parents gave informed consent and the other parent did not object. The study protocol was approved by the medical ethics committee of the UMCG (METc 2011/258).

### Item generation

The instrument was developed using the established method of item generation, item reduction, and construct validation [32]. The purpose of item generation was to assemble all items that affect quality of life of children with BPD at school age. Three sources of information were used: literature, interviews with doctors with clinical experience in follow-up of BPD patients (i.e., neonatologists and pediatric pulmonologists, since they perform the follow-up of the patients in The Netherlands) and interviews with a first cohort of parents of BPD patients. These interviews were semi-structured and completed by one researcher who was trained in interviewing patients. The search was considered to be complete when no new items emerged. This point of saturation was ascertained by the whole research team based on the list of items assembled during literature search, expert interviews and when two consecutive parent interviews elicited no additional items. Additional information on participant selection and the process of item generation is described in Online Resource 1.

### Item reduction

For item reduction, the assembled items were presented to the parents of a second cohort of BPD patients. They indicated on a questionnaire for each item if it was relevant to their child and to what extent on a 6-point numeric rating scale with “0” indicating no burden and “6” extreme burden. With the clinical impact method the overall importance (OI) of each item was determined as the product of the mean importance of an item and the frequency with which it was rated [33, 34]. Items with OI > 1 were selected. Items with high inter-item correlation (IIC > 0.9) were checked for content similarity. The item with the lowest OI was removed unless the research team decided otherwise. Items with low item-total correlation (ITC < 0.2) were discarded [35].

### Construct validation and scale development

In a third cohort of BPD patients, parents were asked to rate the burden of the previously selected items for their child on a questionnaire with a 7-point numeric rating scale ranging from 0 (“no burden”) to 7 (“extreme burden”). Therefore, higher score reflects worse HRQL. To show construct validity, one should use an objective parameter that reflects the severity of the disease but that is not itself a measure of QoL [25]. Five hypotheses were developed to test the construct that children age 4–8 years, who fulfill the criteria for BPD and who experience complications of BPD at this age, have a lower HRQL. These hypotheses can be reviewed in the Online Resource 2. Parameters that are generally accepted to measure health problems of BPD patients and are easily obtained in clinical practice were chosen: FEV_1_, FEV_1_/FVC, and FEF_25–75_ obtained by flow-volume measurement and expressed as *z*-scores [36], body mass index (BMI) in *z*-score [37], mean arterial blood pressure (MAP) in mmHg, the standardized *T*-score of the Dutch version of the age-specific Child Behavior Checklist (CBCL) with addition of the teacher report form (TRF) in children over 6 years old [38, 39], and signs of ventricular hypertrophy by screening of an electrocardiogram (ECG). The outcomes of the tests and the items in the questionnaire were then correlated. Items that correlated at least moderately and also appropriately with one of the hypotheses that define the construct were considered to show construct validity. Items that did not show construct validity were removed from the questionnaire. The remaining items were categorized based on an exploratory factor analysis and face validity [35, 40, 41]. Because of expected sample size, factor analysis was not used to validate the categories but rather to indicate possible categories. Cronbach’s alpha was calculated to estimate internal consistency of the items in the final questionnaire [42]. The instrument was developed in the Dutch language.

### Data analysis

Differences between groups of participants were evaluated with the Student’s *t* Test, Pearson’s Chi-square Test or Fisher’s Exact Test. Differences were considered to be significant when *P* < .05. Correlations were calculated as Spearman’s rho (*ρ*_s_). Correlations were considered high when *ρ*_s_ > 0.50, moderate when *ρ*_s_ = 0.35–0.50 and low when *ρ*_s_ < 0.35. Correlations with *P* < .05 were labeled significant [32]. Statistical analysis was performed with Predictive Analytics Software Statistics 18.0.3.

## Results

### Characteristics of participants

For item generation, 16 parents (94% mothers) of 19 children with BPD and 7 doctors were interviewed. For item reduction parents of 50 children were approached, of which 32 responded. One declined participation because of a language barrier. For clinical evaluation parents of 110 children were approached, of whom 50 responded. Two parents refused participation because they did not want an extra hospital visit for their child, six parents refused for unknown reasons. The characteristics of the three cohorts are documented in Table 1; no significant differences were observed. When reviewing complications experienced during admission to the neonatal intensive care unit, significantly more grade I–II intraventricular hemorrhage occurred in the cohort for validation. The incidence of nephrocalcinosis was very low in the cohort for item reduction, a difference reaching significance when compared to the cohort for validation.

**Table 1:** Population characteristics per study phase

	Item generation	Item reduction	Validation
Characteristics			
Participants	19	31	42
Sex (m/f)	12/7	14/17	27/15
Age in years (mean ± SD)	6.2 ± 1.6	5.5 ± 1.5	5.7 ± 1.4
Multiple births (*n*, %)	9 (47%)	14 (45%)	10 (24%)
Birth weight in grams (mean ± SD)	950 ± 244	951 ± 308	1041 ± 221
Days gestational age at birth (mean ± SD)	193.7 ± 11.0	192.5 ± 8.2	195.0 ± 11.9
Type of BPD*			
Mild (*n*, %)	8 (42%)	9 (29%)	10 (24%)
Moderate (*n*, %)	4 (21%)	13 (42%)	13 (31%)
Severe (*n*, %)	7 (37%)	8 (26%)	17 (41%)
Unknown (*n*, %)	0 (0%)	1 (3%)	2 (5%)
Neonatal complications			
Necrotizing enterocolitis			
Treated conservatively (*n*, %)	0 (0%)	2 (7%)	0 (0%)
Needed surgery (*n*, %)	2 (11%)	0 (0%)	2 (5%)
Persistent ductus arteriosus			
Treated with medication (*n*, %)	8 (42%)	11 (36%)	19 (45%)
Needed surgery (*n*, %)	5 (26%)	7 (23%)	7 (17%)
Intraventricular hemorrhage			
Grade I–II (*n*, %)	1 (5%)	1 (3%)	8 (19%)^‡^
Grade III–IV (*n*, %)	1 (5%)	3 (10%)	0 (0%)
Infection			
Sepsis (*n*, %)	14 (74%)	21 (68%)	27 (64%)
Pneumonia (*n*, %)	3 (16%)	8 (26%)	6 (14%)
Meningitis (*n*, %)	1 (5%)	2 (7%)	1 (2%)
Retinopathy of prematurity			
Grade I–II (*n*, %)	5 (26%)	4 (13%)	7 (17%)
Grade III–V (*n*, %)	1 (5%)	1 (3%)	0 (0%)
Nephrocalcinosis (*n*, %)	6 (32%)	2 (7%)^†^	12 (29%)

*As defined by Jobe and Bancalari in Am J Respir Crit Care Med in 2001 [4]

^†^Significant compared to both identification and clinical impact group (*P* < 0.05)

^‡^Significant compared to clinical impact group (*P* < 0.05)

### Results of item generation and item reduction

A list of 130 problems was assembled and presented to the second cohort of parents of BPD patients for item reduction. The clinical impact method identified 52 problems with an OI > 1. The correlation matrix revealed 6 pairs of problems that had high inter-item correlation. Of three pairs, the one with the lowest overall importance was removed. Two pairs were found to be clinically different and therefore neither item was eliminated. One pair was minimally rephrased to become a single item. There was one item with low item-total correlation which was removed.

### Construct validation

The test parameters that showed relevant correlation with the items in the questionnaire were spirometry (FEV_1_ and FVC), BMI, MAP, and the CBCL including TRF. The FEV_1_/FVC and FEV_25–75_ showed correlation with a few items but all were weak (*ρ*_s_ < 0.308) and none were statistically significant (*P* > .06). No ECG showed signs of ventricular hypertrophy. An overview of the outcome of the tests parameters in the cohort of children in the validation phase is summarized in Table 2. There were 33 items that showed construct validity. All listed items and the correlation coefficients with the tests parameters that support construct validity are shown in Table 3. The correlation coefficients of all items entering validation phase have been added in Online Resource 3. Items in the domain of pulmonary symptoms did not show correlation with the pulmonary function tests but with the results of the TRF. All items concerning school functioning correlated with results of the TRF. The vast majority of items concerning growth and nutrition correlated with BMI, except the items ‘vomiting easily’ which correlated with MAP and ‘stomach ache’ which correlated with the CBCL. The items concerning exercise and locomotion correlated most strongly with pulmonary function results. In this category, the problems focusing on exercise instead of locomotion also correlated significantly with MAP. Items in ‘emotional functioning and health care concerns’ showed correlation with the CBCL and TRF sum scales. Analysis of the item responses show that most of the participants report a burden of 1–2 on the numeric rating scale (between ‘barely’ or ‘a little bit’) and only few participants report the highest burden. Mean responses with floor and ceiling effects are presented in Table 4.

**Table 2:** Outcome of the test parameters in validation phase

Parameter	Number of participants	Value (mean ± SD)	*Z*-score (mean ± SD)
Height (m)	42	1.17 ± 0.10	− 0.72 ± 0.96
Weight (kg)	42	20.91 ± 0.96	− 0.84 ± 1.06
Body Mass Index	42	15.01 ± 1.42	− 0.53 ± 0.88
Mean arterial pressure (mmHg)	42	73.36 ± 9.07	
FEV_1_ (l)	40	1.15 ± 0.31	− 0.88 ± 0.97
FVC (l)	38	1.43 ± 0.39	− 0.11 ± 0.99
FEV_1_/FVC (%)	38	81.86 ± 7.61	− 1.47 ± 0.93
FEV_25–75_ (l)	38	1.18 ± 0.43	− 1.48 ± 1.01
CBCL-TOT (*T*-score)	36	51.33 ± 9.48	
TRF-TOT (*T*-score)	19	48.47 ± 7.37	
TRF-AF (*T*-score)	19	48.53 ± 0.43	

*FEV*_*1*_ forced expiratory volume in 1 s, *FVC* forced vital capacity, *FEV*_*25–75*_ forced expiratory volume at 25–75% of FVC, *CBCL* child behavior checklist, *CBCL-TOT* total scale of CBCL, *TRF* teacher report form, *TRF-TOT* total scale of TRF, *TRF-AF* scale sum of academic functioning of TRF

**Table 3:** Correlation coefficients supporting construct validity for the items in the BPD quality of life questionnaire (BPD-QoL) with the selected measurements and internal consistency of the domains

Items of BPD-QoL	BMI	MAP	FEV_1_	FVC	CBCL-TOT	TRF-TOT	TRF-AF	*α*
Number of participants	42	42	40	38	36	19	19	
Pulmonary symptoms								0.925
Wheezing							0.359	
Sputum in throat or airways						0.402		
Dyspnea when having a cold						0.391	0.359	
Having a cold more often						**0.467**		
Having a cold that lasts longer						**0.588**		
Bronchial hyperreactivity						0.446		
School functioning								0.925
Difficulty progressing in school						**0.621**	0.385	
Needing extra support at school						**0.595**	0.402	
Difficulty concentrating						**0.659**		
Being easily distracted						**0.624**		
Requiring a lot of structure daily						**0.466**		
Difficulty with arithmetic							0.407	
Difficulty with grammar							0.371	
Growth and nutrition								0.845
Stomach ache due to constipation							**0.590**	
Difficulty eating a serving of food	**0.381**							
Taking a long time when eating	**0.395**							
Often don’t want to eat								
Feeling cold easily	**0.419**							
Vomiting easily		**0.422**						
Being sensitive to loud noises	**0.419**							
Exercise and locomotion								0.850
Problems in physical education/sports			**0.430**	**0.542**				
Problems practicing swimming			**0.481**	**0.437**		0.404		
Easily tripping or falling				**0.413**		0.397		
Problems with cycling			**0.468**	**0.424**		0.388		
Problems with coordination or balance			**0.427**	**0.438**				
Exertional dyspnea			**0.360**	**0.419**				
Not keeping up in exercise/playing		**0.385**		**0.367**				
Emotional functioning and health care concerns								0.778
Consults with the general practitioner						0.361	0.454	
Feeling less self-confident						0.406		
Fear of being left alone by parent(s)					**0.369**			
Being shy					**0.453**			
Having scars of surgeries, punctures or intravenous lines						0.452		
Frequent need to use medication						**0.511**		

Significant correlations (*P* < 0.05) are marked in bold

*BMI* body mass index, *MAP* mean arterial pressure, *FEV*_*1*_ forced expiratory volume in 1 s, *FVC* forced vital capacity, *CBCL-TOT* total scale of child behavior checklist, *TRF-TOT* total scale of teacher report form, *TRF-AF* scale sum of academic functioning of teacher report form, *α* Cronbach’s alpha

**Table 4:** Mean, standard deviation, and spread of responses of the 33 final items of BPD-QoL

Items of BPD-QoL	*N*	Mean (SD)	Percentage of participants with no burden	Percentage of participants with highest burden
Coughing	42	2.12 (1.50)	19.0	0.0
Impaction of mucus in airways	41	1.78 (1.29)	19.5	0.0
Sputum in throat or airways	42	1.64 (1.50)	31.0	0.0
Dyspnea when having a cold	42	1.86 (1.69)	33.3	0.0
Having a cold more often	42	1.90 (1.82)	35.7	0.0
Having a cold that lasts longer	42	1.90 (1.82)	35.7	2.4
Bronchial hyperreactivity	41	1.93 (1.88)	36.6	2.4
Having earpain/otitis	42	1.14 (1.26)	42.9	0.0
Difficulty progressing in school	42	1.12 (1.69)	59.5	2.4
Needing extra support at school	42	1.21 (1.69)	57.1	2.4
Difficulty concentrating	42	2.21 (1.87)	28.6	2.4
Being easily distracted	42	2.31 (1.92)	28.6	2.4
Requiring a lot of structure daily	42	2.48 (1.76)	23.8	2.4
Difficulty with arithmetic	42	0.98 (1.66)	71.4	0.0
Difficulty with grammar	41	1.05 (1.70)	68.3	0.0
Difficulty eating a serving of food	42	1.74 (1.70)	35.7	2.4
Taking a long time when eating	42	1.55 (1.70)	42.9	0.0
Often don’t want to eat	42	1.26 (1.55)	47.6	0.0
Feeling cold easily	42	1.55 (1.69)	45.2	0.0
Being sensitive to loud noises	42	1.57 (1.92)	47.6	4.8
Problems in physical education/sports	42	0.81 (1.23)	61.9	0.0
Problems practicing swimming	40	1.20 (1.86)	67.5	0.0
Easily tripping or falling	42	1.52 (1.53)	35.7	2.4
Problems with cycling	42	0.67 (1.05)	66.7	0.0
Problems with coordination or balance	42	1.02 (1.14)	45.2	0.0
Exertional dyspnea	42	1.26 (1.56)	50.0	0.0
Not keeping up in exercise/playing	42	0.95 (1.32)	57.1	0.0
Trouble parting from parent(s)	42	1.12 (1.55)	52.4	0.0
Withdrawal from social situations	42	1.14 (1.35)	50.0	0.0
Consults with the general practitioner	42	0.86 (1.22)	54.8	0.0
Feeling less self-confident	42	1.55 (1.57)	38.1	0.0
Fear of being left alone by parent(s)	42	1.21 (1.62)	50.0	2.4
Being shy	42	1.76 (1.43)	26.2	0.0
Having scars of surgeries, punctures or intravenous lines	42	1.50 (1.57)	40.5	0.0

*N* number of participants, *SD* standard deviation

### Scale development and internal consistency

Principal component analysis with varimax rotation categorized items into six domains [35, 41]. The sixth domain contained only one item (‘stomach ache due to constipation’); therefore, this item was replaced within the domain it fit next best in. Four other items were replaced in a more appropriate domain based on face validity and the pattern of correlation they showed with the test parameters. The five domains were named ‘pulmonary complaints,’ ‘school functioning,’ ‘growth and nutrition,’ ‘exercise and locomotion,’ and ‘emotional functioning and health care concerns.’ The questionnaire showed excellent internal consistency with Cronbach’s alpha of 0.919 and all domains having Cronbach’s alpha exceeding 0.70. Since items have been deleted during the validation process, we chose not to evaluate the internal consistency of the total score. An English translation of the Dutch questionnaire, named BPD-QoL, was added to function as an example in Online Resource 4.

## Discussion

With this study, a Dutch parent-proxy disease-specific quality of life questionnaire for children with BPD aged 4–8 years old (the BPD-QoL) was developed and evaluated for construct validation and internal consistency reliability. This is an important step in developing structured care for these patients since it allows clinicians to aim therapeutic interventions at perceived burdens instead of performing follow-up of the possible physical complications of BPD that might not be relevant to the child’s quality of life.

### Item generation and item reduction

The process of item generation and item reduction was most essential to ensure the development of a complete and relevant questionnaire. Item generation relied not only on existing literature and the opinion of experts, but also on the input from parents of patients. In total, 16 interviews were conducted in a representative sample. This is an adequate amount for item generation [43]. For item reduction, the clinical impact method was used, rather than factor analysis. Literature showed that either method can show different results in questionnaire development, but one is not superior to the other [33, 34]. Since this instrument was developed for clinical practice and clinical research, showing clinical impact was of most importance for items in the questionnaire. Item reduction resulted in an acceptable number of items.

### Process of construct validation

Subsequently, it was determined if the identified items were reliable and valid for the purpose of measuring HRQL [25, 26]. In the absence of a gold standard measurement, construct validity is the best type of validity that can be achieved [25]. Construct validity is based on the concept that items of disease-specific HRQL instruments should be at least moderately correlated to an independent measure of the same disease. This method is established and has successfully been used in the development of HRQL instruments [44]. There is no clinical consensus on how to measure the severity of BPD at school age independently. Therefore, we chose five measurements of functioning that are widely used in the follow-up and determination of complications of BPD [31]. The results of this analysis led to the deletion of 14 items that did not show construct validity. Factor analysis was used to investigate the amount of domains and which items they should contain. The sample size in our study was not sufficient for a reliable exploratory factor analysis and the Kaiser–Meyer–Olkin measure of sampling adequacy was only 0.373 [35, 41]. Factor analysis was therefore used as an indication and the final decision on number of domains and items added to them was made by the research team.

For evaluation of internal consistency of the developed questionnaire and domains, Cronbach’s alpha was determined. This is a measure of scale reliability and the coefficient indirectly estimates to which degree a set of items measures a single construct [42]. The value that Cronbach’s alpha should reach to prove internal consistency of an HRQL instrument is not definite. In literature on scale development and psychometric properties an alpha value from 0.50 to greater than 0.90 can be recommended, depending on the purpose of the instrument [40, 45]. A value > 0.70 is generally accepted to prove good internal consistency in previously developed HRQL instruments [44, 46].

One difficulty in determining construct validity is that selected clinical parameters are not always able to reflect the severity of the problems as experienced by patients. As was also demonstrated in this study, there is lack of correlation between burden of pulmonary symptoms and pulmonary function parameters. This has been shown previously in asthma [47, 48] and chronic cough [49]. In our study, pulmonary function tests show moderate correlation with problems experienced in exercise and locomotion. An ECG can be used as a screening tool for right ventricular hypertrophy as a sign of pulmonary hypertension (PH), even though it is not a very sensitive tool [50]. It showed no value in determining construct validity for the questionnaire since the results showed no signs of PH in our population. In this study, we evaluated MAP as a parameter of cardiovascular functioning. The expectation was that it would be correlated to problems in exercise. Some correlation was found but this was quite modest. The item ‘vomiting easily’ correlated with MAP; however, this is not considered to be a problem in cardiovascular functioning. Otherwise there was not much value for measurement of MAP in determining construct validity. The choice to use the total score of the CBCL was made when we reviewed the correlations and determined that the total score included basically all relevant correlations found in the subscales. Correlations in the total score are however weakened since it contains many subscales. For example, there is weak correlation with problems of withdrawal and lack of self-confidence. When looking at the CBCL subscale on ‘withdrawal,’ stronger correlations are found. The sample size that completed the TRF in our study was small but the correlation with items concerning school functioning suggests that it is a valuable test to evaluate the burden of problems in school functioning in children with BPD. The TRF correlated with many different items in some way, suggesting that these tests explore a large area of functioning of the child. A footnote to the CBCL is that it is designed for children in general and is not specifically validated for the clinical setting.

### Limitations

There were only a few exclusion criteria for the participants. This made it possible to include a considerable number of children and to apply the results of this study to a broad population of children with BPD. On the other hand, comorbidity could have influenced both the test results and the results of the questionnaire. In order to minimize this problem, parents were asked to answer questions with the relationship of the child’s problems to BPD in mind. All children in our study were of low gestational age and low birth weight. The results of the study could partly be caused by solely these conditions without an attributional effect of BPD itself. It is however not of clinical use to make a distinction between these conditions. One could expect some overlap of items when developing a HRQL instrument for children of low gestational age and/or low birth weight; this has not yet been investigated. We considered these conditions to be so much related to BPD that their influence on quality of life should be captured within our developed instrument. Overall, the number of participants in the item reduction and validation phase is small due to the fact that BPD does not have very high prevalence and it is difficult to select a population that does in fact fulfill the set criteria. Also there is not a 100% response rate in the follow-up population. Because item generation was limited by resources and number of participants, no focus groups and no recordings of interviews were collected. Content validity could have been elaborated further by conducting cognitive interviews and collecting qualitative data on item saturation, as is currently recommended in developing patient-reported outcome measures [51]. In the item reduction phase, a satisfactory response rate of 64% was reached. However within the selected population for the validation phase, there was a large group of parents that did not respond to our request to participate or refused to participate (62%). The low response rate could be due to the fact that the research required responders to visit the hospital. Descriptive data from the non-responders were not available. When analyzing descriptive data of the items, we found a substantial portion of our participants reported no burden for the selected items. This can be expected since survivors of BPD are at increased risk of developing complications but not all patients are affected. We consider our included population as a representative sample of the target population. However, from a psychometric point of view, such floor effects are high [52] and could limit the item validity and reliability. Adequate variation remains captured by the items in the BPD-QoL since Cronbach’s alpha is not too high for an HRQL instrument [44], as one would expect when items are too interrelated. Finally, the choice of creating a parent-proxy instrument can be considered to be a limitation since this is not a substitute for an individual’s own information [28, 30]. Although self-report instruments have been developed for children under 8 years old, these show more frequently to be of lower reliability [29, 30]. We found this not to be a valid option in our population when taking into account the possible developmental delay in patients with BPD. Even though large differences can be found when comparing reports of children to their parents on HRQL, parent-proxy measurement of quality of life has shown to be valid [53]. Proxy-reports are likely to be most accurate when items focus on observational content [30], as they do in the BPD-QoL. However, when developing an instrument to measure HRQL in children with BPD above 8 years old, a patient reported outcome measure is recommended [30].

For use in other languages, the Dutch instrument should be translated and validated in that language [54]. The instrument should be evaluated further for convergent and divergent validity, longitudinal validity, reliability, and responsiveness to determine its suitability for use in follow-up and research on HRQL in children with BPD.

## Conclusion

This is the first reported disease-specific instrument to measure parent-proxy HRQL in children 4–8 years old with BPD. It provides a first step in meeting the need of acquiring more accurate measurements of HRQL in this population. Measuring HRQL is useful in determining the long-term effects and evaluating treatment options. The instrument developed in this study showed construct validity and internal consistency but further analysis of reliability and validity is necessary.

## Electronic supplementary material

Below is the link to the electronic supplementary material.

Supplementary material 1 (PDF 66 KB)

Supplementary material 2 (PDF 67 KB)

Supplementary material 3 (PDF 85 KB)

Supplementary material 4 (PDF 582 KB)
