# Supplementary material for: Development and construct validation of a parent-proxy quality of life instrument in children with bronchopulmonary dysplasia aged 4–8 years old
Source: Qual Life Res. 2018 Oct 22;28(2):523–33. doi: 10.1007/s11136-018-2029-7 (PMC6373528; doi:10.1007/s11136-018-2029-7)
Supplement: Supplementary file 1 — Supplementary material 1 (PDF 66 KB) [file 11136_2018_2029_MOESM1_ESM.pdf]

Development and construct validation of a parent-proxy quality of life instrument in children with bronchopulmonary dysplasia aged 4-8 years old

Lysbert Meijer - Schaap[1], MD, Anthony E.J. Dubois[2,3], MD, PhD, Boudewijn J. Kollen[4], PhD, Jet Tijmens – van der Hulst[2], MSc, Bertine M.J. Flokstra – de Blok[3,4], PhD, Elianne J.L.E. Vrijlandt[2,3], MD, PhD

Affiliations: [1]Department of Pulmonary Diseases and Tuberculosis, University of Groningen, University Medical Center Groningen, Groningen, The Netherlands. [2]Department of Pediatrics, Division of Pediatric Pulmonology and Allergy, University of Groningen, University Medical Center Groningen, Groningen, The Netherlands. [3]GRIAC research institute, University of Groningen, University Medical Center Groningen, Groningen, The Netherlands. [4]Department of General Practice, University of Groningen, University Medical Center Groningen, Groningen, The Netherlands.
Corresponding author: L. Meijer – Schaap (l.schaap@umcg.nl).

Additional information on methods of participant selection and item generation

The participants were randomly selected from a database of 239 children with a diagnosis of BPD. They had retrospectively been identified in a pilot study in a cohort of 1061 children that were admitted to the neonatal intensive care unit of the University Medical Center of Groningen between 2003 and 2007[1]. Children were excluded when they had comorbidity not known to be related to BPD but that could lead to similar symptoms or complications like for example symptomatic cardiac disease, congenital malformations and gene disorders. Participants were approached by mail on their home address as registered in the hospital administration. Clinical experts were selected from the network of one of the researchers (Dr. Vrijlandt). They are all working in different hospitals throughout The Netherlands and are pulmonologists and neonatologists active both in BPD care and research. The order of steps in the process of item generation was: 1) literature search, 2) interviews with clinical experts and 3) interviews with parents of patients. In their ability to provide items, all three sources were equally valued. Semi-structured interviews were conducted with parents of patients and clinical experts by using a questionnaire developed by the research team. It consisted of open questions and questions about specific themes that were identified during literature search. Every interview started with an explanation of the study itself and the purpose of the interview. The interview was completed by telephone or by meeting in person with the participant. Notes were made during the interview and these were reviewed afterwards to identify items of quality of life that had not been listed previously. The item list was generated by one researcher (Drs. Meijer - Schaap) and consequently reviewed by the research team to ascertain the point of item saturation and the exact transcription of the items. Item saturation was reached when no new items emerged during two consecutive parent interviews.

[1]. De Roest JG. Bronchopulmonale dysplasie: de huidige incidentie en de gevolgen op de algemene en pulmonale ontwikkeling in de eerste twee levensjaren [abstract]. 2009; http://irs.ub.rug.nl/dbi/4b868ce55f9af.
