# Supplementary material for: Development and construct validation of a parent-proxy quality of life instrument in children with bronchopulmonary dysplasia aged 4–8 years old
Source: Qual Life Res. 2018 Oct 22;28(2):523–33. doi: 10.1007/s11136-018-2029-7 (PMC6373528; doi:10.1007/s11136-018-2029-7)
Supplement: Supplementary file 2 — Supplementary material 2 (PDF 67 KB) [file 11136_2018_2029_MOESM2_ESM.pdf]

Development and construct validation of a parent-proxy quality of life instrument in children with bronchopulmonary dysplasia aged 4-8 years old

Lysbert Meijer - Schaap[1], MD, Anthony E.J. Dubois[2,3], MD, PhD, Boudewijn J. Kollen[4], PhD, Jet Tijmens – van der Hulst[2], MSc, Bertine M.J. Flokstra – de Blok[3,4], PhD, Elianne J.L.E. Vrijlandt[2,3], MD, PhD

Affiliations: [1]Department of Pulmonary Diseases and Tuberculosis, University of Groningen, University Medical Center Groningen, Groningen, The Netherlands. [2]Department of Pediatrics, Division of Pediatric Pulmonology and Allergy, University of Groningen, University Medical Center Groningen, Groningen, The Netherlands. [3]GRIAC research institute, University of Groningen, University Medical Center Groningen, Groningen, The Netherlands. [4]Department of General Practice, University of Groningen, University Medical Center Groningen, Groningen, The Netherlands.
Corresponding author: L. Meijer – Schaap (l.schaap@umcg.nl).

Construct of the BPD quality of life questionnaire:
Children age 4 to 8 years old, who fulfill the criteria for BPD and who experience complications of BPD at this age, have lower HRQL.

Hypotheses to test construct validity:

1. Children who experience more pulmonary function abnormalities, as a complication of BPD, have a lower HRQL.

1.1 A negative correlation is expected between FEV1, FEV1/FVC, FEF25-75 score and BPD-QL scores.
1.2 This correlation is expected to be moderate or high with the items concerning pulmonary complaints and low with the other items.

2. Children who have low height, weight and/or BMI according to their age, as a complication of BPD, have lower HRQL.

2.1 A negative correlation is expected between length, weight, BMI and BPD-QL scores.
2.2 This correlation is expected to be moderate or high with the items concerning growth and nutrition and low with the other items.

3. Children who experience signs of pulmonary or systemic hypertension, as a complication of BPD, have lower HRQL.

3.1 A positive correlation between existence of ventricular hypertrophy and BPD-QL scores and between the MAP and BPD-QL scores is expected.
3.2 This correlation is expected to be moderate or high with the items concerning cardiovascular functioning and low with the other items.

4. Children who experience more learning disabilities, as a complication of BPD, have lower HRQL.

4.1 A negative correlation is expected between the CBCL scores on scales of the teacher report form and

BPD-QL scores.

4.2 This correlation is expected to be moderate or high with the items concerning school functioning and low with the other items.

5. Children who experience more problems with behavior and emotional functioning, as a complication of BPD, have lower HRQL.

5.1 A positive correlation between the CBCL scores on scales of behavior and emotional functioning and BPD-QL scores is expected.

5.2 This correlation is expected to be moderate or high with the items concerning emotional functioning and low with the other items.
