# Supplementary material for: Development and construct validation of a parent-proxy quality of life instrument in children with bronchopulmonary dysplasia aged 4–8 years old
Source: Qual Life Res. 2018 Oct 22;28(2):523–33. doi: 10.1007/s11136-018-2029-7 (PMC6373528; doi:10.1007/s11136-018-2029-7)
Supplement: Supplementary file 3 — Supplementary material 3 (PDF 85 KB) [file 11136_2018_2029_MOESM3_ESM.pdf]

Development and construct validation of a parent-proxy quality of life instrument in children with bronchopulmonary dysplasia aged 4-8 years old

Lysbert Meijer - Schaap[1], MD, Anthony E.J. Dubois[2,3], MD, PhD, Boudewijn J. Kollen[4], PhD, Jet Tijmens – van der Hulst[2], MSc, Bertine M.J. Flokstra – de Blok[3,4], PhD, Elianne J.L.E. Vrijlandt[2,3], MD, PhD

Affiliations: [1]Department of Pulmonary Diseases and Tuberculosis, University of Groningen, University Medical Center Groningen, Groningen, The Netherlands. [2]Department of Pediatrics, Division of Pediatric Pulmonology and Allergy, University of Groningen, University Medical Center Groningen, Groningen, The Netherlands. [3]GRIAC research institute, University of Groningen, University Medical Center Groningen, Groningen, The Netherlands. [4]Department of General Practice, University of Groningen, University Medical Center Groningen, Groningen, The Netherlands.

Corresponding author: L. Meijer – Schaap (l.schaap@umcg.nl).

Correlation coefficients of all items entering validation phase with the selected measurements.

**Items**	**BMI**	**MAP**	**FEV_1**	**FVC**	**CBCL -TOT**	**TRF-TOT**	**TRF-AF**
**Number of participants**	**42**	**42**	**40**	**38**	**36**	**19**	**19**
Coughing	.125	.130	-.116	-.071	.138	.322	-.221
Wheezing	.024	.098	.109	.110	.180	.085	-.359
Impaction of mucus in airways	.084	.099	-.071	-.057	.153	.099	.132
Sputum in throat or airways	.034	.195	-.147	-.085	.036	.402	-.047
Dyspnea when having a cold	.022	.221	-.127	.029	.180	.391	-.359
Having attacks of dyspnea	.013	.149	-.057	-.079	.120	.101	-.307
Having a cold more often	.070	.124	.045	.034	.169	**.467**	-.208
Having a cold that lasts longer	-.029	.176	.057	.051	.081	**.588**	-.349
Bronchial hyperreactivity	.055	.101	-.017	.089	.222	.446	-.294
Having earpain/otitis	.108	.173	-.084	.111	.028	.188	-.031
Trouble sleeping because of dyspnea	.014	.245	-.106	-.131	.042	.193	-.221
Trouble sleeping because of coughing	.109	.112	-.061	-.098	.082	.258	-.139
Need more sleep than other children	-.236	.021	-.124	-.276	.066	-.008	.010
Difficulty progressing in school	.052	.085	.123	.104	.242	**.621**	-.385
Needing extra support at school	.124	.164	.051	.104	.288	**.595**	-.402
Difficulty concentrating	.125	-.062	.206	.158	.219	**.659**	-.230
Being easily distracted	.113	-.056	.186	.155	.162	**.624**	-.234
Requiring a lot of structure daily	.072	.106	.078	.116	.318	**.466**	-.250
Difficulty with arithmetic	.006	**.315**	.143	.183	.053	.314	-.407
Difficulty with grammar	-.034	.227	-.032	-.060	-.063	.327	-.371
Choking easily	-.197	.038	-.077	-.277	.137	.136	-.224
Stomach ache due to constipation	-.069	-.171	.160	.156	**.335**	.337	**-.590**
Difficulty eating a serving of food	**-.381**	.275	-.065	-.146	.109	.236	-.060
Taking a long time when eating	**-.395**	.200	-.055	-.146	.140	-.139	.118
Often don't want to eat	**-.331**	.242	-.021	-.124	.192	.083	-.120
Feeling cold easily	**-.419**	.083	.064	-.062	-.040	-.183	.196
Vomiting easily	.023	**.422**	-.294	-.291	-.054	-.365	**.464**
Being sensitive to loud noises	**-.419**	-.074	-.114	-.186	.236	.041	.159
Problems with writing neatly	.090	.069	-.285	-.115	-.051	.223	.424
Problems in physical education/sports	-.024	.319	**-.430**	**-.542**	-.324	.014	.445
Problems practicing swimming	-.148	.250	**-.481**	**-.437**	-.193	.404	.141
Easily tripping or falling	-.056	-.015	-.296	**-.413**	.086	.397	.226
Problems with cycling	.018	.198	**-.468**	**-.424**	-.108	.388	**.487**
Problems with coordination or balance	.007	.041	**-.427**	**-.438**	-.101	.212	**.638**
Exertional dyspnea	-.063	**.328**	**-.360**	**-.419**	-.137	.242	.004
Easily tired during sports/exercise	-.027	**.314**	-.270	-.260	-.148	.234	.244
Not keeping up in exercise/playing	-.101	**.385**	**-.339**	**-.367**	-.193	.404	.141
Trouble parting from parent(s)	.180	.292	.058	.043	.294	-.119	-.058
Withdrawal from social situations	-.187	-.005	-.111	-.186	.153	.317	-.170
Consults with the general practitioner	-.016	-.011	-.044	.035	.321	.361	-.454
Feeling less self-confident	-.076	.302	-.118	-.139	.299	.406	-.109
Fear of being left alone by parent(s)	.203	.039	.120	.107	**.369**	-.065	-.012
Being shy	.067	.081	.181	.159	**.453**	.119	-.240
Having scars of surgeries, punctures or intravenous lines	.053	.079	-.114	-.039	.279	.452	-.242
Frequent need to use antibiotics	.022	.053	-.146	-.065	.210	.112	-.058
Frequent need to use airway medication	-.006	-.310	-.241	**.318**	-.062	.221	-.302
Frequent need to use medication	-.060	.153	-.271	-.209	.077	**.511**	-.229

Significant correlations ($P<0.05$) are marked bold. Abbreviations: BMI= body mass index; MAP= mean arterial pressure; FEV_1= Forced Expiratory Volume in 1 second; FVC= Forced Vital Capacity; CBCL-TOT= total scale of child behavior checklist; TRF-TOT= total scale of teacher report form, TRF-AF= scale sum of academic functioning of teacher report form, α= Cronbach's alpha.
