# Supplementary material for: Development and construct validation of a parent-proxy quality of life instrument in children with bronchopulmonary dysplasia aged 4–8 years old
Source: Qual Life Res. 2018 Oct 22;28(2):523–33. doi: 10.1007/s11136-018-2029-7 (PMC6373528; doi:10.1007/s11136-018-2029-7)
Supplement: Supplementary file 4 — Supplementary material 4 (PDF 582 KB) [file 11136_2018_2029_MOESM4_ESM.pdf]

Development and construct validation of a parent-proxy quality of life instrument in children with bronchopulmonary dysplasia aged 4-8 years old

Lysbert Meijer - Schaap[1], MD, Anthony E.J. Dubois[2,3], MD, PhD, Boudewijn J. Kollen[4], PhD, Jet Tijmens – van der Hulst[2], MSc, Bertine M.J. Flokstra – de Blok[3,4], PhD, Elianne J.L.E. Vrijlandt[2,3], MD, PhD

Affiliations: [1]Department of Pulmonary Diseases and Tuberculosis, University of Groningen, University Medical Center Groningen, Groningen, The Netherlands. [2]Department of Pediatrics, Division of Pediatric Pulmonology and Allergy, University of Groningen, University Medical Center Groningen, Groningen, The Netherlands. [3]GRIAC research institute, University of Groningen, University Medical Center Groningen, Groningen, The Netherlands. [4]Department of General Practice, University of Groningen, University Medical Center Groningen, Groningen, The Netherlands.
Corresponding author: L. Meijer – Schaap (l.schaap@umcg.nl).

Example of the BPD-QoL (4-8 years): a parent-proxy questionnaire to measure health-related quality of life in children with bronchopulmonary dysplasia aged 4 to 8 years old. Please note that this English translation has not been validated.

Instructions
This questionnaire is developed to determine the influence of bronchopulmonary dysplasia on the quality of life of your child. Please indicate for each question the amount of burden this item has on the quality of life of your child in your opinion. Please encircle one of the presented seven options:

0 = None
1 = Barely
2 = A little bit
3 = Fairly
4 = Quite
5 = Very
6 = Extremely

We ask you kindly to try to answer the questions in the way that you believe that your child would answer these. The questionnaire contains two pages.

BPD-QoL (4-8 years): a questionnaire to measure health-related quality of life in children with bronchopulmonary dysplasia aged 4 to 8 years old.

		None	Barely	A little bit	Fairly	Quite	Very	Extremely
**Pulmonary symptoms**<br>**What is the amount of burden your child experiences with...**								
1	Pulmonary wheezing?	0	1	2	3	4	5	6
2	Having sputum in throat or airways?	0	1	2	3	4	5	6
3	Being short of breath when having a cold?	0	1	2	3	4	5	6
4	Having a cold more often than other children?	0	1	2	3	4	5	6
5	Having a cold that lasts longer than in other children?	0	1	2	3	4	5	6
6	Having complaints of bronchial hyperreactivity?	0	1	2	3	4	5	6
**School functioning**<br>**What is the amount of burden your child experiences with...**								
7	Having difficulty progressing in school?	0	1	2	3	4	5	6
8	Needing extra support at school?	0	1	2	3	4	5	6
9	Having difficulty concentrating?	0	1	2	3	4	5	6
10	Being easily distracted?	0	1	2	3	4	5	6
11	Needing a lot of structure in daily life?	0	1	2	3	4	5	6
12	Having difficulty with arithmetic?	0	1	2	3	4	5	6
13	Having difficulty with grammar?	0	1	2	3	4	5	6

BPD-QoL (4-8 years): a questionnaire to measure health-related quality of life in children with bronchopulmonary dysplasia aged 4 to 8 years old.

		None	Barely	A little Bit	Fairly	Quite	Very	Extremely
**Growth and nutrition**<br>**What is the amount of burden your child experiences with...**								
14	Having stomach ache due to constipation?	0	1	2	3	4	5	6
15	Having difficulty eating a normal serving of food?	0	1	2	3	4	5	6
16	Taking a long time when eating?	0	1	2	3	4	5	6
17	Often disliking to eat food?	0	1	2	3	4	5	6
18	Feeling cold easily?	0	1	2	3	4	5	6
19	Vomiting easily?	0	1	2	3	4	5	6
20	Being sensitive to loud noises?	0	1	2	3	4	5	6
**Exercise and locomotion**<br>**What is the amount of burden your child experiences with...**								
21	Having difficulty in physical education or sports?	0	1	2	3	4	5	6
22	Having difficulty in practicing swimming?	0	1	2	3	4	5	6
23	Easily tripping or falling?	0	1	2	3	4	5	6
24	Having difficulty cycling?	0	1	2	3	4	5	6
25	Having difficulty with coordination and balance?	0	1	2	3	4	5	6
26	Having shortness of breath when exercising?	0	1	2	3	4	5	6
27	Not being able to keep up with other children in running or playing?	0	1	2	3	4	5	6
**Emotional functioning and health care concerns**<br>**What is the amount of burden your child experiences with...**								
28	Having to consult the general practitioner?	0	1	2	3	4	5	6
29	Feeling less self confident?	0	1	2	3	4	5	6
30	Having fear of being left alone by his/her parent(s)?	0	1	2	3	4	5	6
31	Being shy?	0	1	2	3	4	5	6
32	Having scars of surgeries, punctions or lines that were necessary during hospitalization?	0	1	2	3	4	5	6
33	Frequently needing to use medication?	0	1	2	3	4	5	6
